# Multi-gene phylogenetic evidence indicates that *Pleurodesmospora* belongs in Cordycipitaceae (Hypocreales, Hypocreomycetidae) and *Pleurodesmospora lepidopterorum* sp. nov. on pupa from China

**DOI:** 10.3897/mycokeys.80.66794

**Published:** 2021-05-07

**Authors:** Wan-Hao Chen, Yan-Feng Han, Jian-Dong Liang, Wei-Yi Tian, Zong-Qi Liang

**Affiliations:** 1 Basic Medical School, Guizhou University of Traditional Chinese Medicine, Guiyang 550025, Guizhou, China Guizhou University of Traditional Chinese Medicine Guiyang China; 2 Institute of Fungus Resources, Department of Ecology, College of Life Sciences, Guizhou University, Guiyang 550025, Guizhou, China Guizhou University Guiyang China

**Keywords:** Insect, morphological characteristic, new species, phylogenetic analysis, taxonomic placement

## Abstract

A new species, *Pleurodesmospora lepidopterorum*, isolated from a pupa, is introduced. Morphological comparisons and phylogenetic analyses based on multigene datasets (ITS+RPB1+RPB2+TEF) support the establishment of the new species. *Pleurodesmospora lepidopterorum* is distinguished from *P. coccorum* by its longer conidiogenous pegs located in the terminal or lateral conidiophores, and smaller subglobose or ellipsoidal conidia. A combined dataset of RPB1, RPB2, and TEF confirmed the taxonomic placement of *Pleurodesmospora* in Cordycipitaceae for the first time.

## Introduction

The genus *Pleurodesmospora* was established for the type species *P. coccorum* (Petch) Samson, W. Gams & H.C. Evans (Samson et al. 1980). The typical characteristic of *Pleurodesmospora* is its erect or procumbent conidiophores, which bear numerous minute phialidic conidiogenous pegs in the terminal or mostly intercalary position, often in whorls below the septa. Conidiogenous pegs are short-cylindrical and give rise to short chains of conidia. Conidia are ellipsoid to dacryoid with a slightly truncate base (Samson et al. 1980).

*Pleurodesmospora* species have diverse ecological characteristics, and have been found on scale insects, whitefly, aphids, leaf-hoppers, spider and scavenger mites (Petch 1931; Samson and McCoy 1982; Samson et al. 1980). Li et al. (1991) reported *Pleurodesmospora* as a newly recorded genus in China and confirmed for the first time that *P. coccorum* has strong pathogenicity to black whitefly. According to Index Fungorum, the taxonomic status of *Pleurodesmospora* is incertae sedis.

During a survey of entomopathogenic fungi from Southwest China, a new insect-associated species was found. The morphological characteristics of the new species resembled *Pleurodesmospora*. In our phylogenetic analyses of combined RPB1, RPB2 and TEF sequences, *Pleurodesmospora* clustered in Cordycipitaceae (Hypocreales, Hypocreomycetidae) with strong statistical support and was closely related to *Beauveria* Vuill. and *Akanthomyces* Lebert. Thus, we propose that *Pleurodesmospora* belongs to family Cordycipitaceae and introduce *Pleurodesmospora lepidopterorum* sp. nov. as a new insect-associated species on the basis of morphological comparison and molecular phylogenetic analyses.

## Materials and methods

### Specimen collection and identification

An infected pupa of Lepidoptera specimen (DY1050) was collected from Duyun City (26°21'24.71"N, 107°22'48.22"E), Qiannan Buyi and Miao Autonomous Prefecture, Guizhou Province, on 1 October 2019. Isolation of strains was conducted as described by Chen et al. (2019). Fungal colonies emerging from specimens were isolated and cultured at 25 °C for 14 days under 12 h light/12 h dark conditions following protocols described by Zou et al. (2010). Specimens and the isolated strains were deposited in the Institute of Fungus Resources, Guizhou University (formally Herbarium of Guizhou Agricultural College; code, GZAC), Guiyang City, Guizhou, China.

Macroscopic and microscopic morphological characteristics of the fungi were examined and the growth rates were determined from potato dextrose agar (PDA) and oatmeal agar (OA) cultures incubated at 25 °C for 14 days. Hyphae and conidiogenous structures were mounted in lactophenol cotton blue or 20% lactate solution and observed with an optical microscope (OM, DM4 B, Leica, Germany).

### DNA extraction, polymerase chain reaction amplification and nucleotide sequencing

DNA extraction was carried out by Fungal genomic DNA Extraction Kit (DP2033, BioTeke Corporation) in accordance with Liang et al. (2011). The extracted DNA was stored at −20 °C. The internal transcribed spacer (ITS) region, RNA polymerase II largest subunit 1 (RPB1), RNA polymerase II largest subunit 2 (RPB2) and translation elongation factor 1 alpha (TEF) were amplified by PCR as described by White et al. (1990), Castlebury et al. (2004) and van den Brink et al. (2004), respectively. PCR products were purified and sequenced at Sangon Biotech (Shanghai) Co. The resulting sequences were submitted to GenBank.

### Sequence alignment and phylogenetic analyses

Lasergene software (version 6.0, DNASTAR) was applied for the assembling and editing of DNA sequence. The ITS, RPB1, RPB2 and TEF sequences were downloaded from GenBank, based on Mongkolsamrit et al. (2018, 2020) and others selected on the basis of BLAST algorithm-based searches in GenBank (Table 1). The multiple datasets of ITS, RPB1, RPB2 and TEF were aligned and edited by MAFFT v7.037b (Katoh and Standley 2013) and MEGA6 (Tamura et al. 2013). Assembling of the combined datasets (RPB1+RPB2+TEF and ITS+RPB1+RPB2+TEF) was performed by SequenceMatrix v.1.7.8 (Vaidya et al. 2011). The model was selected for Bayesian analysis by ModelFinder (Kalyaanamoorthy et al. 2017) in the software PhyloSuite (Zhang et al. 2020).

**Table 1.** Taxa included in the phylogenetic analyses.

Species	Strain No.	GenBank accession No.			
		ITS	RPB1	RPB2	TEF
*Akanthomyces aculeatus*	HUA 186145	–	–	–	MF416465
	HUA 772	KC519371	–	–	KC519366
*Akanthomyces attenuates*	CBS 402.78	–	EF468888	EF468935	EF468782
*Akanthomyces lecanii*	CBS 101247	–	DQ522407	DQ522466	DQ522359
*Akanthomyces waltergamsii*	TBRC 7251	–	MF140781	MF140805	MF140833
	TBRC 7252		MF140782	MF140806	MF140834
*Ascopolyporus polychrous*	P.C. 546	–	DQ127236	–	DQ118745
*Ascopolyporus villosus*	ARSEF 6355	AY886544	DQ127241	–	DQ118750
*Beauveria bassiana*	ARSEF 1564	HQ880761	HQ880833	HQ880905	HQ880974
	ARSEF 7518	HQ880762	HQ880834	HQ880906	HQ880975
*Beauveria brongniartii*	ARSEF 617	–	HQ880854	HQ880926	HQ880991
*Beauveria caledonica*	ARSEF 2567	–	HQ880889	HQ880961	EF469057
*Blackwellomyces cardinalis*	OSC 93609	–	DQ522370	DQ522370	DQ522325
	OSC 93610	JN049843	EF469088	EF469106	EF469059
*Claviceps purpurea*	S.A. cp11	–	EF469087	EF469105	EF469058
*Clonostachys rosea*	AFTOL ID.187	–	–	DQ862029	–
	GJS 90227	–	–	–	AY489611
*Conoideocrella luteorostrata*	NHJ 11343	–	EF468906	–	EF468801
	NHJ 12516	–	EF468905	EF468946	EF468800
*Cordyceps kyusyuensis*	EFCC 5886	–	EF468863	–	EF468754
*Cordyceps militaris*	OSC 93623	JN049825	DQ522377	–	DQ522332
*Cordyceps ninchukispora*	E.G.S.38.165	–	EF468900	–	EF468795
	E.G.S.38.166	–	EF468901	–	EF468794
*Cordyceps piperis*	CBS 116719	–	DQ127240	EU369083	DQ118749
*Gibellula gamsii*	BCC 25798	MH152532	EU369056	EU369076	EU369018
	BCC 27968	MH152529	MH152547	–	MH152560
*Hevansia novoguineensis*	CBS 610.80	MH532831	–	MH521844	MH521885
	NHJ 11923	–	EU369052	EU369072	EU369013
*Hyperdermium pulvinatum*	P.C. 602	–	DQ127237	–	DQ118746
*Lecanicillium antillanum*	CBS 350.85	MH861888	DQ522396	DQ522450	DQ522350
*Lecanicillium psalliotae*	CBS 101270	–	EF469096	EF469112	EF469067
	CBS 532.81	–	EF469095	EF469113	EF469066
*Lecanicllium tenuipes*	CBS 309.85	–	DQ522387	DQ522439	DQ522341
*Metarhizium anisopliae*	ARSEF 7487	–	DQ468355	DQ468370	DQ463996
	CBS 130.71	–	MT078861	MT078918	MT078845
*Metarhizium flavoviride*	CBS 125.65	–	MT078862	MT078919	MT078846
	CBS 700.74	–	MT078863	MT078920	MT078847
*Neotorrubiella chinghridicola*	BCC 39684	–	MK632071	MK632181	MK632148
	BCC 80733	–	MK632072	MK632176	MK632149
*Ophiocordyceps gracilis*	EFCC 8572	–	EF468859	EF468912	EF468751
*Ophiocordyceps sinensis*	EFCC 7287	–	EF468874	EF468924	EF468767
*Orbiocrella petchii*	NHJ 6209	–	EU369061	EU369081	EU369023
*Pleurodesmospora coccorum*	CBS 458.73	MH860741	–	–	–
	CBS 459.73	MH860742	–	–	–
	CBS 460.73	MH860743	–	–	–
***Pleurodesmospora lepidopterorum***	**DY10501**	**MW826576**	**MW834315**	**MW834316**	**MW834317**
	**DY10502**	**MW826577**	–	**MW834318**	**MW834319**
*Polycephalomyces formosus*	ARSEF 1424	–	DQ127245	KF049671	DQ118754
*Polycephalomyces paracuboideus*	NBRC 101742	–	KF049647	KF049669	KF049685
*Purpureocillium lilacinum*	ARSEF 2181	–	EF468896	–	EF468790
	CBS 431.87	–	EF468897	EF468940	EF468791
*Purpureocillium lilacinum*	CBS 284.36	MH855800	EF468898	EF468941	EF468792
*Samsoniella aurantia*	TBRC 7271	–	MF140791	–	MF140846
	TBRC 7272	MF140763	–	MF140817	MF140845
*Simplicillium lanosoniveum*	CBS 101267	–	DQ522405	DQ522463	DQ522357
	CBS 704.86	AJ292396	DQ522406	DQ522464	DQ522358
*Yosiokobayasia kusanagiensis*	TNS–F18494	–	JN049890	–	JF416014

The datasets (RPB1+RPB2+TEF and ITS+RPB1+RPB2+TEF) were analyzed by Bayesian inference (BI) and maximum likelihood (ML) methods to determine the relationship among *Pleurodesmospora* and related genera in the order Hypocreales (analysis 1) and the relationship among *Pleurodesmospora* and related genera in the family Cordycipitaceae (analysis 2), respectively. For BI, a Markov chain Monte Carlo (MCMC) algorithm was used to generate phylogenetic trees with Bayesian probabilities using MrBayes v.3.2 (Ronquist et al. 2012) for the combined sequence datasets. The Bayesian analysis resulted in 20,001 trees after 10,000,000 generations. The first 4,000 trees, representing the burn-in phase of the analyses, were discarded, while the remaining 16,001 trees were used for calculating posterior probabilities in the majority rule consensus tree. After the analysis was finished, each run was examined using the program Tracer v1.5 (Drummond and Rambaut 2007) to determine burn-in and confirm that both runs had converged. ML analyses were constructed with RAxMLGUI (Silvestro et al. 2012). The GTRGAMMA model was used for all partitions, in accordance with recommendations in the RAxML manual against the use of invariant sites.

## Results

### Phylogenetic analyses

**Figure 1.**
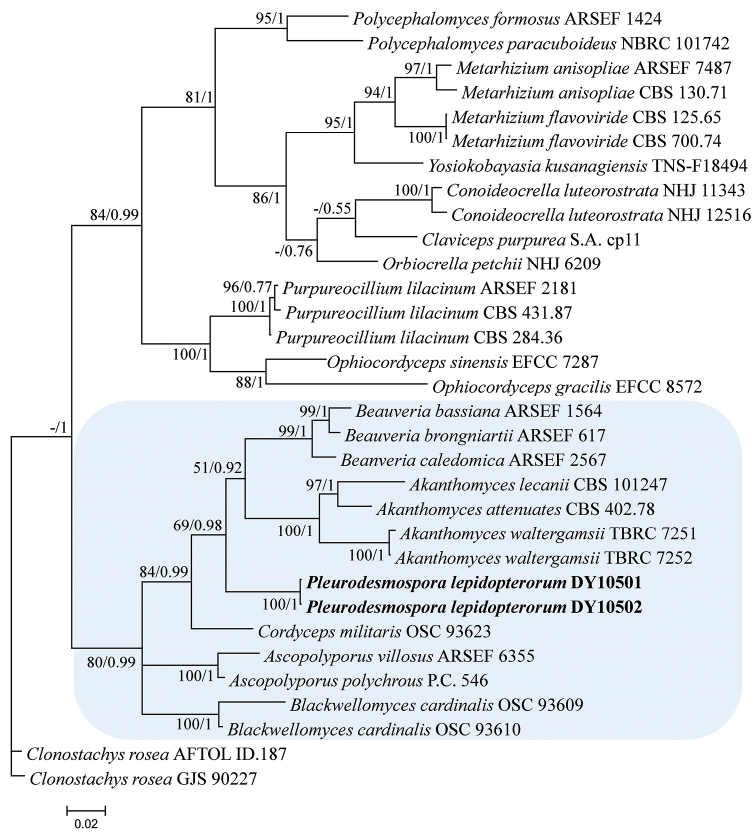
Phylogenetic relationships among *Pleurodesmospora* and related genera in the order Hypocreales based on a multigene dataset (RPB1, RPB2, and TEF). Statistical support values (≥ 50%/0.5) are shown at the nodes for maximum likelihood bootstrap support/ Bayesian inference posterior probabilities.

**Figure 2.**
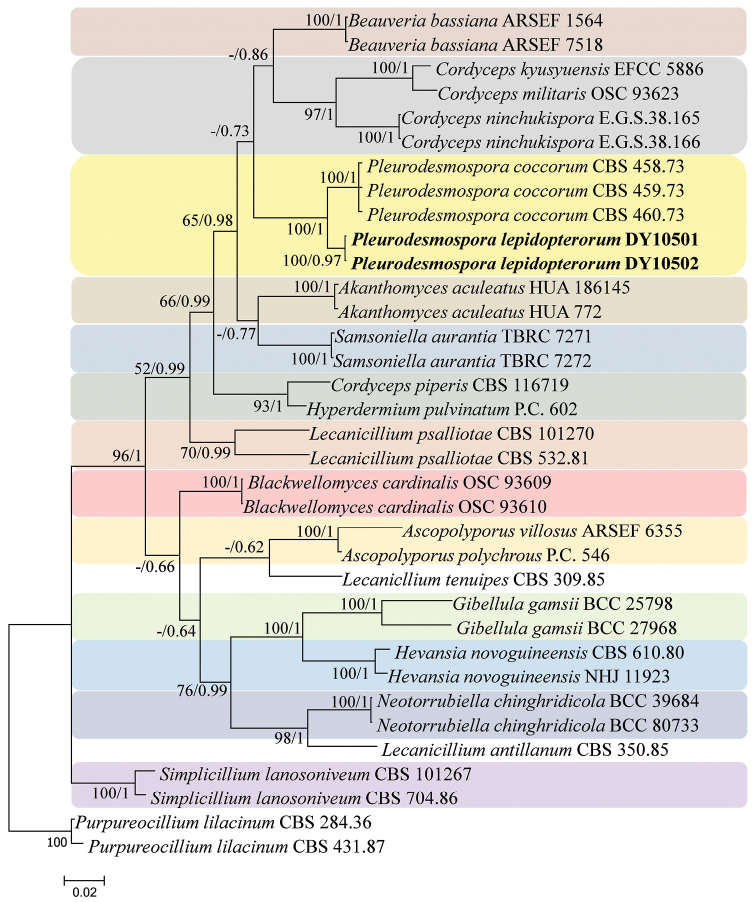
Phylogenetic relationships among *Pleurodesmospora* and related genera in the family Cordycipitaceae based on a multigene dataset (ITS, RPB1, RPB2 and TEF). Statistical support values (≥ 50%/0.5) are shown at the nodes for maximum likelihood bootstrap support/Bayesian inference posterior probabilities.

*Clonostachys rosea* (Link) Schroers, Samuels, Seifert & W. Gams isolates (AFTOL ID.187 and GJS 90227) were used as the outgroup in analysis 1 (Fig. 1), and *Purpureocillium lilacinum* (Thom) Luangsa-ard, Houbraken, Hywel-Jones & Samson isolates (CBS 284.36 and CBS 431.87) were used as the outgroup in analysis 2 (Fig. 2). The concatenated sequences of analysis 1 and 2 included 23 and 21 taxa, respectively, and consisted of 2,262 (RPB1, 561; RPB2, 821; and TEF, 880) and 2,711 (ITS, 597; RPB1, 508; RPB2, 852; and TEF, 754) characters with gaps, respectively.

Analysis 1: The final value of the highest scoring tree was –18,860.236896, which was obtained from the ML analysis of the dataset (RPB1+RPB2+TEF). The parameters of GTR model to analysis of the dataset were estimated base frequencies; A = 0.240138, C = 0.290732, G = 0.262224, T = 0.206905; substitution rates AC = 1.004710, AG = 3.103423, AT = 0.837508, CG = 0.886482, CT = 5.821155, GT = 1.000000; gamma distribution shape parameter α = 0.309925. The selected model for BI analysis were K2P+G4 (RPB2) and GTR+F+I+G4 (RPB1+TEF). In the order-level phylogenetic tree (Fig. 1), the maximum likelihood and Bayesian inference trees were generally congruent, and most branches were strongly supported. The new strains clustered with the genera *Cordyceps*, *Akanthomyces*, and *Beauveria*, and belonged to family Cordycipitaceae.

Analysis 2: The final value of the highest scoring tree was –19,321.404482, which was obtained from the ML analysis of the dataset (ITS+RPB1+RPB2+TEF). The parameters of GTR model to analysis of the dataset were estimated base frequencies; A = 0.238334, C = 0.298168, G = 0.261443, T = 0.202055; substitution rates AC = 0.963749, AG = 2.807654, AT = 0.822463, CG = 0.766574, CT = 5.738062, GT = 1.000000; gamma distribution shape parameter α = 0.339059. The selected model for BI analysis were HKY+F+G4 (ITS) and GTR+F+I+G4 (RPB1+RPB2+TEF). In the family-level phylogenetic tree (Fig. 2), the maximum likelihood and Bayesian inference trees were generally congruent, and most branches were strongly supported. The new strains formed an independent branch but clustered with *Pleurodesmospora coccorum*; therefore, these strains represent a new species described as *P. lepidopterorum*.

### Taxonomy

#### Pleurodesmospora lepidopterorum

Taxon classificationFungiHypocrealesCordycipitaceae

W.H. Chen, Y.F. Han & Z.Q. Liang sp. nov.

10F80ED8-145D-5185-B29C-D112B174E23E

839148

Figure 3

##### Diagnosis.

Differs from *P. coccorum* by having longer conidiogenous pegs located in the terminal or lateral conidiophores, and smaller subglobose or ellipsoidal conidia.

##### Type.

China, Guizhou Province, Qiannan Buyi and Miao Autonomous Prefecture, Duyun City (26°21'24.71"N, 107°22'48.22"E), 1 October 2019, Wanhao Chen, holotype GZAC DY1050, ex-type culture GZAC DY10501. Sequences from isolated strain DY10501 have been deposited in GenBank with accession numbers: ITS = MW826576, RPB1 = MW834315, RPB2 = MW834316 and TEF = MW834317.

##### Description.

Colonies on PDA, 3.9–4.1 cm diam. in 14 d at 25 °C, white, consisting of a basal felt and cottony, floccose hyphal overgrowth, reverse pale yellowish. Prostrate hyphae smooth, septate, hyaline, 1.3–1.9 μm diam. Erect or procumbent conidiophores usually arising from aerial hyphae, barely differentiated from vegetative hyphae, usually branched. Conidiogenous cells polyphialidic, terminal and intercalary, bearing numerous short-cylindrical, 1.8–3.5 μm long and 0.7–1.3 μm wide conidiogenous pegs, in whorls often below the septa. The terminal or lateral conidiogenous cells cylindrical, 5.9–12.0 × 1.8–2.2 μm. Conidia in chains, hyaline, smooth-walled, subglobose or ellipsoidal, one-celled, 2.3–3.6 × 1.7–3.3 μm. Chlamydospores and synnemata not observed. Size and shape of phialides and conidia similar in culture on PDA, OA agar and on natural substrate. Sexual state not observed.

**Figure 3.**
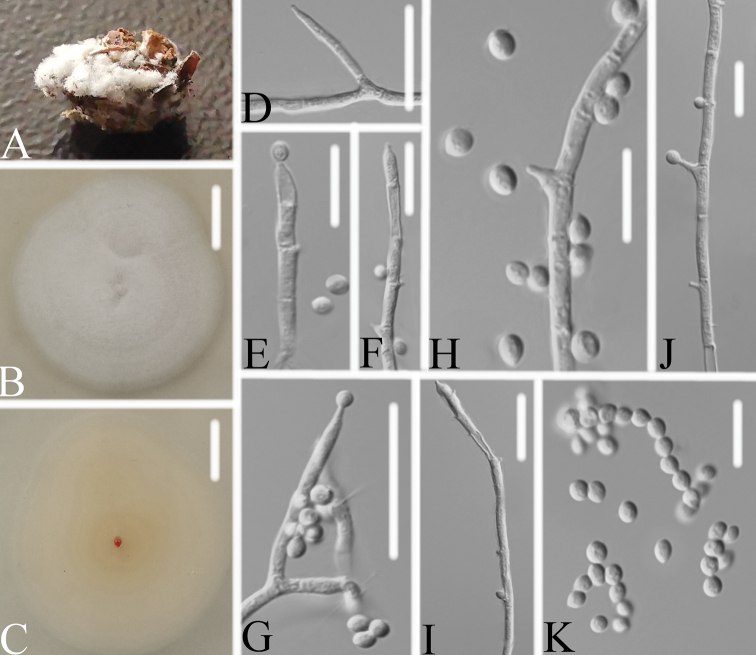
*Pleurodesmospora lepidopterorum***A** infected pupa (Lepidoptera) **B, C** top (**B**) and underside (**C**) of a colony cultured on PDA medium at 14 d **D–J** conidiogenous pegs and conidia **K** conidia in chains. Scale bars: 10 mm (**B, C**) 10 μm (**D–K**).

##### Host.

Pupa, order Lepidoptera.

##### Distribution.

Duyun City, Qiannan Buyi and Miao Autonomous Prefecture, Guizhou Province, China.

##### Etymology.

Referring to its insect host, which belongs to order Lepidoptera.

##### Remarks.

*Pleurodesmospora lepidopterorum* was readily identified as belonging to *Pleurodesmospora* in the family-level phylogenetic tree (Fig. 2). When compared with the typical characteristics of *P. coccorum*, *P. lepidopterorum* was easily distinguished by its longer conidiogenous pegs located in the terminal or lateral conidiophores, and smaller subglobose or ellipsoidal conidia.

## Discussion

BLAST results of ITS, RPB1, RPB2, and TEF sequence data revealed that the strain DY10501 was similar to several taxa in GenBank: ITS, 98.62% similar to *Lecanicillium* sp. (isolate ICMP:20146); RPB1, 88.55% similar to *Beauveria caledonica* Bissett & Widden (isolate ARSEF 7117); RPB2, 86.53% similar to *Cordyceps* sp. (isolate A12116); TEF, 95.33% similar to *Beauveria bassiana* (Bals.-Criv.) Vuill. (isolate CHE-CNRCB 82). In the family-level phylogenetic tree, strains DY10501 and DY10502 formed an independent branch and clustered with *P. coccorum* in a subclade.

Samson et al. (1980) introduced the genus *Pleurodesmospora* with *P. coccorum*, but the taxonomic status of the genus was unclear. Unfortunately, *P. coccorum* lacked RPB1, RPB2, and TEF sequences in GenBank. Therefore, *P. lepidopterorum* was used for multigene analysis of *Pleurodesmospora* and related genera in the order Hypocreales. In the order-level phylogenetic tree, *P. lepidopterorum* clustered into Cordycipitaceae (Hypocreales, Hypocreomycetidae, Sordariomycetes). Thus, the combined dataset of RPB1, RPB2, and TEF confirmed the taxonomic placement of *Pleurodesmospora* in Cordycipitaceae for the first time.

## Supplementary Material

XML Treatment for Pleurodesmospora lepidopterorum

## Supplementary materials

Supplementary material 1 Dataset for Figure 1 Data type molecular data Explanation note A dataset of RPB1, RPB2 and TEF for Figure 1. https://binary.pensoft.net/file/540174This dataset is made available under the Open Database License (http://opendatacommons.org/licenses/odbl/1.0/). The Open Database License (ODbL) is a license agreement intended to allow users to freely share, modify, and use this Dataset while maintaining this same freedom for others, provided that the original source and author(s) are credited.Wan-Hao Chen, Yan-Feng Han, Jian-Dong Liang, Wei-Yi Tian, Zong-Qi Liang

Supplementary material 2 Dataset for Figure 2 Data type molecular data Explanation note A dataset of ITS, RPB1, RPB2 and TEF for Figure 2 https://binary.pensoft.net/file/540175This dataset is made available under the Open Database License (http://opendatacommons.org/licenses/odbl/1.0/). The Open Database License (ODbL) is a license agreement intended to allow users to freely share, modify, and use this Dataset while maintaining this same freedom for others, provided that the original source and author(s) are credited.Wan-Hao Chen, Yan-Feng Han, Jian-Dong Liang, Wei-Yi Tian, Zong-Qi Liang

Supplementary material 3 Table S1. Taxa included in the phylogenetic analyses Data type molecular data https://binary.pensoft.net/file/540176This dataset is made available under the Open Database License (http://opendatacommons.org/licenses/odbl/1.0/). The Open Database License (ODbL) is a license agreement intended to allow users to freely share, modify, and use this Dataset while maintaining this same freedom for others, provided that the original source and author(s) are credited.Wan-Hao Chen, Yan-Feng Han, Jian-Dong Liang, Wei-Yi Tian, Zong-Qi Liang
