# Supplementary material for: Multi-gene phylogenetic evidence indicates that Pleurodesmospora belongs in Cordycipitaceae (Hypocreales, Hypocreomycetidae) and Pleurodesmospora lepidopterorum sp. nov. on pupa from China
Source: MycoKeys. 2021 May 7;80:45–55. doi: 10.3897/mycokeys.80.66794 (PMC8124063; doi:10.3897/mycokeys.80.66794)
Supplement: Supplementary material 3 — Table S1. Taxa included in the phylogenetic analyses [file mycokeys-80-045-s003.docx]

| Species | Strain No. | GenBank accession No. | | | |
| --- | --- | --- | --- | --- | --- |
|  |  | ITS | RPB1 | RPB2 | TEF |
| *Akanthomyces aculeatus* | HUA 186145 | - | - | - | MF416465 |
| *Akanthomyces aculeatus* | HUA 772 | KC519371 | - | - | KC519366 |
| *Akanthomyces attenuates* | CBS 402.78 | - | EF468888 | EF468935 | EF468782 |
| *Akanthomyces lecanii* | CBS 101247 | - | DQ522407 | DQ522466 | DQ522359 |
| *Akanthomyces waltergamsii* | TBRC 7251 | - | MF140781 | MF140805 | MF140833 |
| *Akanthomyces waltergamsii* | TBRC 7252 |  | MF140782 | MF140806 | MF140834 |
| *Ascopolyporus polychrous* | P.C. 546 | - | DQ127236 | - | DQ118745 |
| *Ascopolyporus villosus* | ARSEF 6355 | AY886544 | DQ127241 | - | DQ118750 |
| *Beanveria caledomica* | ARSEF 2567 | - | HQ880889 | HQ880961 | EF469057 |
| *Beauveria bassiana* | ARSEF 1564 | HQ880761 | HQ880833 | HQ880905 | HQ880974 |
| *Beauveria bassiana* | ARSEF 7518 | HQ880762 | HQ880834 | HQ880906 | HQ880975 |
| *Beauveria brongniartii* | ARSEF 617 | - | HQ880854 | HQ880926 | HQ880991 |
| *Blackwellomyces cardinalis* | OSC 93609 | - | DQ522370 | DQ522370 | DQ522325 |
| *Blackwellomyces cardinalis* | OSC 93610 | JN049843 | EF469088 | EF469106 | EF469059 |
| *Claviceps purpurea* | S.A. cp11 | - | EF469087 | EF469105 | EF469058 |
| *Clonostachys rosea* | AFTOL ID.187 | - | - | DQ862029 | - |
| *Clonostachys rosea* | GJS 90227 | - | - | - | AY489611 |
| *Conoideocrella luteorostrata* | NHJ 11343 | - | EF468906 | - | EF468801 |
| *Conoideocrella luteorostrata* | NHJ 12516 | - | EF468905 | EF468946 | EF468800 |
| *Cordyceps kyusyuensis* | EFCC 5886 | - | EF468863 | - | EF468754 |
| *Cordyceps militaris* | OSC 93623 | JN049825 | DQ522377 | - | DQ522332 |
| *Cordyceps ninchukispora* | E.G.S.38.165 | - | EF468900 | - | EF468795 |
| *Cordyceps ninchukispora* | E.G.S.38.166 | - | EF468901 | - | EF468794 |
| *Cordyceps piperis* | CBS 116719 | - | DQ127240 | EU369083 | DQ118749 |
| *Gibellula gamsii* | BCC 25798 | MH152532 | EU369056 | EU369076 | EU369018 |
| *Gibellula gamsii* | BCC 27968 | MH152529 | MH152547 | - | MH152560 |
| *Hevansia novoguineensis* | CBS 610.80 | MH532831 | - | MH521844 | MH521885 |
| *Hevansia novoguineensis* | NHJ 11923 | - | EU369052 | EU369072 | EU369013 |
| *Hyperdermium pulvinatum* | P.C. 602 | - | DQ127237 | - | DQ118746 |
| *Lecanicillium antillanum* | CBS 350.85 | MH861888 | DQ522396 | DQ522450 | DQ522350 |
| *Lecanicillium psalliotae* | CBS 101270 | - | EF469096 | EF469112 | EF469067 |
| *Lecanicillium psalliotae* | CBS 532.81 | - | EF469095 | EF469113 | EF469066 |
| *Lecanicllium tenuipes* | CBS 309.85 | - | DQ522387 | DQ522439 | DQ522341 |
| *Metarhizium anisopliae* | ARSEF 7487 | - | DQ468355 | DQ468370 | DQ463996 |
| *Metarhizium anisopliae* | CBS 130.71 | - | MT078861 | MT078918 | MT078845 |
| *Metarhizium flavoviride* | CBS 125.65 | - | MT078862 | MT078919 | MT078846 |
| *Metarhizium flavoviride* | CBS 700.74 | - | MT078863 | MT078920 | MT078847 |
| *Neotorrubiella chinghridicola* | BCC 39684 | - | MK632071 | MK632181 | MK632148 |
| *Neotorrubiella chinghridicola* | BCC 80733 | - | MK632072 | MK632176 | MK632149 |
| *Ophiocordyceps gracilis* | EFCC 8572 | - | EF468859 | EF468912 | EF468751 |
| *Ophiocordyceps sinensis* | EFCC 7287 | - | EF468874 | EF468924 | EF468767 |
| *Orbiocrella petchii* | NHJ 6209 | - | EU369061 | EU369081 | EU369023 |
| *Pleurodesmospora coccorum* | CBS 458.73 | MH860741 | - | - | - |
| *Pleurodesmospora coccorum* | CBS 459.73 | MH860742 | - | - | - |
| *Pleurodesmospora coccorum* | CBS 460.73 | MH860743 | - | - | - |
| ***Pleurodesmospora lepidopterorum*** | **DY10501** | **MW826576** | **MW834315** | **MW834316** | **MW834317** |
| ***Pleurodesmospora lepidopterorum*** | **DY10502** | **MW826577** | **-** | **MW834318** | **MW834319** |
| *Polycephalomyces formosus* | ARSEF 1424 | - | DQ127245 | KF049671 | DQ118754 |
| *Polycephalomyces paracuboideus* | NBRC 101742 | - | KF049647 | KF049669 | KF049685 |
| *Purpureocillium lilacinum* | ARSEF 2181 | - | EF468896 | - | EF468790 |
| *Purpureocillium lilacinum* | CBS 431.87 | - | EF468897 | EF468940 | EF468791 |
| *Purpureocillium lilacinum* | CBS 284.36 | MH855800 | EF468898 | EF468941 | EF468792 |
| *Samsoniella aurantia* | TBRC 7271 | - | MF140791 | - | MF140846 |
| *Samsoniella aurantia* | TBRC 7272 | MF140763 | - | MF140817 | MF140845 |
| *Simplicillium lanosoniveum* | CBS 101267 | - | DQ522405 | DQ522463 | DQ522357 |
| *Simplicillium lanosoniveum* | CBS 704.86 | AJ292396 | DQ522406 | DQ522464 | DQ522358 |
| *Yosiokobayasia kusanagiensis* | TNS-F18494 | - | JN049890 | - | JF416014 |
